# Disseminating research findings: what should researchers do? A systematic scoping review of conceptual frameworks

**DOI:** 10.1186/1748-5908-5-91

**Published:** 2010-11-22

**Authors:** Paul M Wilson, Mark Petticrew, Mike W Calnan, Irwin Nazareth

**Affiliations:** 1 Centre for Reviews and Dissemination, University of York, YO10 5DD, UK; 2 Social and Environmental Health Department, London School of Hygiene and Tropical Medicine, WC1E 7HT, UK; 3 School of Social Policy, Sociology and Social Research, University of Kent, CT2 7NF, UK; 4 MRC General Practice Research Framework, University College London, NW1 2ND, UK

## Abstract

**Background:**

Addressing deficiencies in the dissemination and transfer of research-based knowledge into routine clinical practice is high on the policy agenda both in the UK and internationally.

However, there is lack of clarity between funding agencies as to what represents dissemination. Moreover, the expectations and guidance provided to researchers vary from one agency to another. Against this background, we performed a systematic scoping to identify and describe any conceptual/organising frameworks that could be used by researchers to guide their dissemination activity.

**Methods:**

We searched twelve electronic databases (including MEDLINE, EMBASE, CINAHL, and PsycINFO), the reference lists of included studies and of individual funding agency websites to identify potential studies for inclusion. To be included, papers had to present an explicit framework or plan either designed for use by researchers or that could be used to guide dissemination activity. Papers which mentioned dissemination (but did not provide any detail) in the context of a wider knowledge translation framework, were excluded. References were screened independently by at least two reviewers; disagreements were resolved by discussion. For each included paper, the source, the date of publication, a description of the main elements of the framework, and whether there was any implicit/explicit reference to theory were extracted. A narrative synthesis was undertaken.

**Results:**

Thirty-three frameworks met our inclusion criteria, 20 of which were designed to be used by researchers to guide their dissemination activities. Twenty-eight included frameworks were underpinned at least in part by one or more of three different theoretical approaches, namely persuasive communication, diffusion of innovations theory, and social marketing.

**Conclusions:**

There are currently a number of theoretically-informed frameworks available to researchers that can be used to help guide their dissemination planning and activity. Given the current emphasis on enhancing the uptake of knowledge about the effects of interventions into routine practice, funders could consider encouraging researchers to adopt a theoretically-informed approach to their research dissemination.

## Background

Healthcare resources are finite, so it is imperative that the delivery of high-quality healthcare is ensured through the successful implementation of cost-effective health technologies. However, there is growing recognition that the full potential for research evidence to improve practice in healthcare settings, either in relation to clinical practice or to managerial practice and decision making, is not yet realised. Addressing deficiencies in the dissemination and transfer of research-based knowledge to routine clinical practice is high on the policy agenda both in the UK [1-5] and internationally [6].

As interest in the research to practice gap has increased, so too has the terminology used to describe the approaches employed [7,8]. Diffusion, dissemination, implementation, knowledge transfer, knowledge mobilisation, linkage and exchange, and research into practice are all being used to describe overlapping and interrelated concepts and practices. In this review, we have used the term dissemination, which we view as a key element in the research to practice (knowledge translation) continuum. We define dissemination as a planned process that involves consideration of target audiences and the settings in which research findings are to be received and, where appropriate, communicating and interacting with wider policy and health service audiences in ways that will facilitate research uptake in decision-making processes and practice.

Most applied health research funding agencies expect and demand some commitment or effort on the part of grant holders to disseminate the findings of their research. However, there does appear to be a lack of clarity between funding agencies as to what represents dissemination [9]. Moreover, although most consider dissemination to be a shared responsibility between those funding and those conducting the research, the expectations on and guidance provided to researchers vary from one agency to another [9].

We have previously highlighted the need for researchers to consider carefully the costs and benefits of dissemination and have raised concerns about the nature and variation in type of guidance issued by funding bodies to their grant holders and applicants [10]. Against this background, we have performed a systematic scoping review with the following two aims: to identify and describe any conceptual/organising frameworks designed to be used by researchers to guide their dissemination activities; and to identify and describe any conceptual/organising frameworks relating to knowledge translation continuum that provide enough detail on the dissemination elements that researchers could use it to guide their dissemination activities.

## Methods

The following databases were searched to identify potential studies for inclusion: MEDLINE and MEDLINE In-Process and Other Non-Indexed Citations (1950 to June 2010); EMBASE (1980 to June 2010); CINAHL (1981 to June 2010); PsycINFO (1806 to June 2010); EconLit (1969 to June 2010); Social Services Abstracts (1979 to June 2010); Social Policy and Practice (1890 to June 2010); Cochrane Database of Systematic Reviews, Cochrane Central Register of Controlled Trials, Cochrane Methodology Register, Database of Abstracts of Reviews of Effects, Health Technology Assessment Database, NHS Economic Evaluation Database (Cochrane Library 2010: Issue 1).

The search terms were identified through discussion by the research team, by scanning background literature, and by browsing database thesauri. There were no methodological, language, or date restrictions. Details of the database specific search strategies are presented Additional File 1, Appendix 1.

Citation searches of five articles [11-15] identified prior to the database searches were performed in Science Citation Index (Web of Science), MEDLINE (OvidSP), and Google Scholar (February 2009).

As this review was undertaken as part of a wider project aiming to assess the dissemination activity of UK applied and public health researchers [16], we searched the websites of 10 major UK funders of health services and public health research. These were the British Heart Foundation, Cancer Research UK, the Chief Scientist Office, the Department of Health Policy Research Programme, the Economic and Social Research Council (ESRC), the Joseph Rowntree Foundation, the Medical Research Council (MRC), the NIHR Health Technology Assessment Programme, the NIHR Service Delivery and Organisation Programme and the Wellcome Trust. We aimed to identify any dissemination/communication frameworks, guides, or plans that were available to grant applicants or holders.

We also interrogated the websites of four key agencies with an established record in the field of dissemination and knowledge transfer. These were the Agency for Healthcare Research and Quality *(*AHRQ*)*, the Canadian Institutes of Health Research (CIHR), the Canadian Health Services Research Foundation (CHSRF), and the Centre for Reviews and Dissemination (CRD).

As a number of databases and websites were searched, some degree of duplication resulted. In order to manage this issue, the titles and abstracts of records were downloaded and imported into EndNote bibliographic software, and duplicate records removed.

References were screened independently by two reviewers; those studies that did not meet the inclusion criteria were excluded. Where it was not possible to exclude articles based on title and abstract alone, full text versions were obtained and their eligibility was assessed independently by two reviewers. Where disagreements occurred, the opinion of a third reviewer was sought and resolved by discussion and arbitration by a third reviewer.

To be eligible for inclusion, papers needed to either present an explicit framework or plan designed to be used by a researcher to guide their dissemination activity, or an explicit framework or plan that referred to dissemination in the context of a wider knowledge translation framework but that provided enough detail on the dissemination elements that a researcher could then use it. Papers that referred to dissemination in the context of a wider knowledge translation framework, but that did not describe in any detail those process elements relating to dissemination were excluded from the review. A list of excluded papers is included in Additional File 2, Appendix 2.

For each included paper we recorded the publication date, a description of the main elements of the framework, whether there was any reference to other included studies, and whether there was an explicit theoretical basis to the framework. Included papers that did not make an explicit reference to an underlying theory were re-examined to determine whether any implicit use of theory could be identified. This entailed scrutinising the references and assessing whether any elements from theories identified in other papers were represented in the text. Data from each paper meeting the inclusion criteria were extracted by one researcher and independently checked for accuracy by a second.

A narrative synthesis [17] of included frameworks was undertaken to present the implicit and explicit theoretical basis of included frameworks and to explore any relationships between them.

## Results

**Figure 1:**
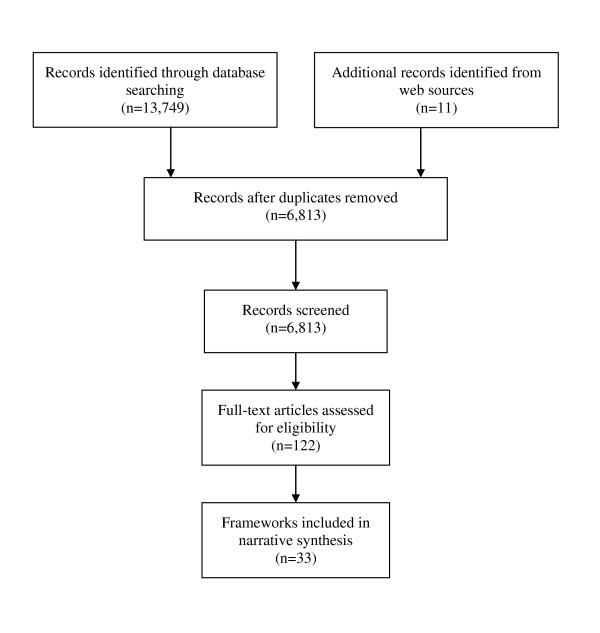
**Identification of conceptual frameworks**.

Our searches identified 6,813 potentially relevant references (see Figure 1). Following review of the titles and abstracts, we retrieved 122 full papers for a more detailed screening. From these, we included 33 frameworks (reported in 44 papers) Publications that did not meet our inclusion criteria are listed in Additional File 2, Appendix 2.

### Characteristics of conceptual frameworks designed to be used by researchers

Table 1 summarises in chronological order, twenty conceptual frameworks designed for use by researchers [11,14,15,18-34]. Where we have described elements of frameworks that have been reported across multiple publications, these are referenced in the Table.

**Table 1:** Conceptual frameworks designed for use by researchers

Author, Year, Aims	Dissemination elements	Theoretical foundations	Description/Comment
Winkler [11] 1985 Develop a model to aid understanding about how new medical information in general and technology assessments in particular reaches practising physician and affects their practice	The source of communication The channels of communication The communication message The characteristics of the audience receiving the communication The setting in which the communication is received	**Persuasive communication** Explicitly based on McGuire's five attributes of persuasive communication. **Diffusion of innovations** Also sets framework in the context specifically the innovation-decision process. **Reference to other included frameworks** None	Communication effectiveness determined by five attributes. Appears to be first application of McGuire's matrix to the context of medical technology assessment. Argues that formal information dissemination followed by informal interaction with influential and knowledgeable colleagues likely to have most impact.

CRD [17,18] 1994, 2009 Presents a framework to be used by researchers seeking to promote the findings of a systematic review.	Review topic Message Audience Source Setting/context Communication channels Implementation of strategy Feed back and evaluation	**Persuasive communication** Revised version acknowledges McGuire's five attributes of persuasive communication. Implicit in original version that is explicitly derived from Winkler. **Diffusion of innovations** 2009 version also sets framework in the context of Diffusion of innovations specifically the innovation-decision process. **Reference to other included frameworks** Winkler Lomas Greenhalgh in 2009 version Hughes in 2009 version Lavis in 2009 version	Framework for disseminating the findings of systematic reviews. Originally postulated that dissemination effectiveness influenced by the sources of communications, media used, and audiences targeted. Later versions acknowledge other elements of persuasive communications and expand into a three phase 'plan, develop, and implement process that assumes interaction with target audiences and consideration of setting in which messages received.

National Center for the Dissemination of Disability Research (NCDDR)[19,38] 1996, 2001 To provide a knowledge base for strengthening the ways in which research results can be accessed and used by those who need them.	*source (i.e.*, agency, organization, or individual responsible for creating the new knowledge or product, and/or for conducting dissemination activities) *content *(message that is disseminated, that is, the new knowledge or product itself, as well as any supporting information or materials) *medium *(*i.e*., ways in which the knowledge or product is described, 'packaged,' and transmitted) *user (*or intended user, of the information or product to be disseminated)	**Persuasive communication** Not explicitly stated but four (source, message, audience, channel) of McGuire's five attributes of persuasive communication evident. **Diffusion of innovations** Also mentions Diffusion of Innovations; specifically the innovation-decision process. **Reference to other included frameworks** None	Review of literature suggests that some combination of four major dimensions of knowledge utilization that can help to strengthen dissemination efforts. A detailed practical ten step-by-step guide for researchers later produced.

Hughes [20,60] 2000 Review the process of dissemination by those who carry it out, those who disseminate it and those who, potentially, make use of it. Examine current approaches to dissemination, considered their effectiveness, highlight obstacles to successful integration of research into practice, and suggest a range of strategies to assist successful dissemination and implementation of research findings.	Provide accessible summaries of research Keep the research report brief and concise Publish in journals or publications which are user friendly Use language and styles of presentation which engage interest Target the material to the needs of the audience Extract the policy and practice implications of research Tailor dissemination events to the target audience and evaluate them Use the media Use a combination of dissemination methods Be proactive Understand external factors	**Persuasive communication** Not explicitly stated but four (setting, message, audience, channel) of McGuire's five attributes of persuasive communication evident. **Reference to other included frameworks** CRD	Commissioned by the Joseph Rowntree Foundation, a framework based on non-systematic literature review and survey of key informants and organisations (including CRD). Authors suggest that active dissemination of research is often under resourced by research commissioners and researchers and that insufficient time and money are set aside when the original funding is considered Five factors identified as contributing to effective dissemination: relevance, quality, accessibility, ownership and timing. List for researchers of factors that can help them disseminate research successfully. Report also outlines suggestions for commissioners, policy makers and practitioners for improving the effectiveness of research dissemination.

Harmsworth [21] 2001 To help educational development projects engaged in the dissemination of new products, materials and good practice in learning and teaching to create an effective dissemination strategy	What is dissemination? What do we want to disseminate? Who are our stakeholders and what are we offering them? When do we disseminate? What are the most effective ways of disseminating? Who might help us disseminate? How do we prepare our strategy? How do we turn our strategy into an action plan? How do we cost our dissemination activities? How do we know we have been successful?	**Persuasive communication** Not explicitly stated but three (message, audience, channel) of the McGuire's five attributes of persuasive communication evident **Reference to other included frameworks** None	Practical question based guide for educational development projects. States that it is based on experiences from over 100 educational development projects, in particular, the Fund for the Development of Teaching and Learning (FDTL) and the Teaching, Learning Technology Programme (TLTP) and Innovations Fund.

Herie [22] 2002 Presents an integrated dissemination model for social work and case study example to illustrate the practical application of the model	Assess market opportunities and identify target system Engage target system Field test the intervention Disseminate the intervention broadly Gather system feedback and provide ongoing consultation.	**Diffusion of innovations** **Social marketing** **Reference to other included frameworks** NCDDR	Describes an integrated dissemination model for social work and provides an example to illustrate its practical application (OutPatient Treatment In ONtario Services -OPTIONS project) Argues that diffusion of innovations and social marketing address the important question of how to put the products of research where they will do the most good: into the hands of practicing clinicians.

Scullion [23] 2002 Examine examples of effective dissemination strategies, provide insights and suggest pointers for researchers, research students and others who may be involved in dissemination.	Source of the message Message characteristics Medium selected to present the message Target users	**Persuasive communication** Not explicitly stated but four (message, source, audience, channel) of McGuire's five attributes of persuasive communication **Reference to other included frameworks** Carpenter CRD Lavis	Practical guide aimed at nursing researchers. Refers to early descriptions of the CRD approach [39]. Author argues that current commitment evidence-based practice will have limited impact on practice and patient care until a similar commitment to dissemination is evident at both corporate and individual levels.

Jacobson [14] 2003 To develop a framework that researchers and other knowledge disseminators who are embarking on knowledge translation can use to increase their familiarity with the intended user groups.	Five domains: The user group The issue The research The researcher-user relationship Dissemination strategies	**None stated** **Reference to other included frameworks** None	Novel framework derived from a review of the research utilisation literature and from the authors' own experience. Emphasises the importance of understanding user context. Each 'domain' provides researchers with a set of questions that can be used to aid the prioritisation of audiences and to develop and tailor relevant messages across user groups.

Lavis [15] 2003 Provide an organizing framework for a knowledge transfer strategy and an overview of our understanding of the current knowledge for each of the five elements of the framework	What should be transferred to decision makers? To whom should it be transferred? By whom should research knowledge be transferred? How should research knowledge be transferred? With what effect should research knowledge be transferred?	**Persuasive communication** Not explicitly stated but four (message, audience, source, channel) of McGuire's five attributes of persuasive communication **Reference to other included frameworks** None	Organising framework and overview of literature relating to knowledge transfer strategies. Question format implicitly mirrors Lasswell's famous description of the act of communications as 'Who says what in which channel to whom with what effect' [37].

Farkas [24] 2003 Describe a conceptual framework for the dissemination and utilisation of information, long with examples of its use	Exposure strategies are those dissemination methods that focus on the goal of increased knowledge Experience strategies focus on the goal of increased positive attitudes towards the new knowledge Expertise strategies focus on the goal of increased competence Embedding strategies target consumers tend to be personally focused	**Diffusion of innovations** Diffusion of innovations in that research has concluded knowledge is not a 'thing to be sent and received. Rather disseminating new findings or information involves communicating through 'certain channels over time among members of a social system' **Reference to other included frameworks** NCDDR	Authors suggest most dissemination practices are not organized or planned to achieve comprehensive impact. Role of framework is to help researchers understand dissemination and utilization as a series of active learning strategies and to direct these at particular knowledge goals and the needs of particular users. Paper also presents examples of '4E' use.

Economic and Social Research Council [26] 2004 Provide advice on planning and prioritising activities and includes a template you can use to structure your own strategy. Aimed at research directors but is applicable to any communications exercise and should be useful to a wider group of researchers.	Checking perceptions Setting objectives Agreeing principles Developing messages and branding Prioritising audiences Choosing channels Planning activities Estimating time Estimating budget Evaluating success	**Persuasive communication** Not explicitly stated but four (message, audience, source as branding, channel) of McGuire's five attributes of persuasive communication **Reference to other included frameworks** None	A detailed practical step-by-step guide on planning and prioritising research communication. Involves all key elements of McGuire's persuasive communication matrix but also addresses more practical issues such as timing and availability of resources. Available at: www.esrc.ac.uk/ESRCInfoCentre/CTK/communications-strategy/default.aspx

Canadian Health Services Research Foundation [25] 2004 List of Key elements that should be included in a dissemination plan. Provide a good overview of some of the most critical things that should be considered	Project overview Dissemination goals Target audiences Key messages (contextualised) Sources/messengers Dissemination activities, tools, timing and responsibilities Budget Evaluation	**Persuasive communication** Not explicitly stated but all (message, audience, setting, source, channel) of McGuire's five attributes of persuasive communication **Reference to other included frameworks** None	Brief overview of key elements that should be considered as part of a collaborative research planning process. Involves all key elements of McGuire's persuasive communication matrix but also addresses more practical issues such as timing and availability of resources. Available at: www.chsrf.ca/keys/use_disseminating_e.php

European Commission [27] 2004 Aims to assist project coordinators and team leaders to generate an effective flow of information and publicity about the objectives and results of their work, the contributions made to European knowledge and scientific excellence, the value of collaboration on a Europe-wide scale, and the benefits to EU citizens in general.	Defining key messages Establishing target audiences Selecting the appropriate modes of communication Tailoring information to the intended outlets Building good relationships with the media Evaluating results Maximising the exposure of messages Tapping useful Commission and other external resources	**Persuasive communication** Not explicitly stated but three (message, audience, channel) of McGuire's five attributes of persuasive communication **Reference to other included frameworks** None	Practical guide aimed at researchers in EU Sixth (now seventh) Framework Programme projects. Provides an outline of good practices to assist researchers to generate an effective flow of information and publicity about the objectives and results of their work. Focuses primarily on research communication via mass media channels

Carpenter [28] 2005 Designed to assist the Agency for Healthcare Research and Quality (AHRQ) Patient Safety grantees with disseminating their research results	What is going to be disseminated? Who will apply it in practice? Through whom can you reach end users? How you convey the research outcomes? How you determine what worked? Where do you start?	**Persuasive communication** Not explicit but four (message, audience, source, channel) of McGuire's five attributes of persuasive communication derived from Lavis **Diffusion of innovations** **Reference to other included frameworks** NCDDR Lavis	Practical guide including six major elements aimed at AHRQ patient safety researchers. Basic premise is to provide a structure to what can be a nebulous concept yet which researchers are increasingly expected to respond. Emphasises importance of engaging end users in planning process.

Bauman [29] 2006 Provide a six step framework for understanding international approaches to physical activity diffusion and dissemination.	Describe the innovation, its rationale and evidence base, and its relevance in an international context; Describe the target audience for dissemination and the sequence, timing, and formatting of dissemination strategies; Define the international communication channels for the innovation; Determine the role of key policymakers and sustainable partnerships that are needed to implement the innovation at different levels (local, state, national, international); Identify the barriers and facilitators of the innovation in the international context; and Conduct research and evaluation to understand the dissemination process.	**Diffusion of innovations** Application of Diffusion of Innovations in a public health context **Persuasive communication** Not explicitly stated but three (audience, channel, setting) of McGuire's five attributes of persuasive communication **Reference to other included frameworks** None	Authors emphasise that dissemination one part of diffusion process. Much of framework based on expert opinion and experiences. Four case studies presented to illustrate aspects of framework. Authors suggest that these share some common elements, including strong advocacy, good communications between key individuals and institutions, and the presence of shared values and population-level approaches.

Zarinpoush [31] 2007 To provide a framework that is intended to help non-profit organizations plan, conduct, and evaluate efforts to transfer and exchange knowledge with others	Define the target audience Preparing the message (Clear, Concise, Consistent, Compelling, Continuous) Selection of transfer method (s) Messenger credibility Evaluation of expected effects	**Persuasive communication** Not explicitly stated but four (message, source, audience, channel) of McGuire's five attributes of persuasive communication **Reference to other included frameworks** Lavis	Five key elements to consider when planning knowledge transfer and exchange activity. States elements derived from recent literature, including Lavis.

Formoso [30] 2007 To analyse the barriers to knowledge transfer that are often inherent in the format of the information communicated. Proposes a more user-friendly, enriched format to facilitate the translation of evidence-based information into practice.	Five dimensions for enhancing information delivery: Contextualization/enrichment Validity/critical appraisal Comprehensibility of data on clinical benefits and harms Applicability and relevance Straightforwardness and appeal	**Social marketing** **Reference to other included frameworks** None	Describes five dimensions for enhancing information delivery and argues that little attention is focussed on the way clinical information is constructed and communicated and how it can be made more relevant, acceptable and eventually 'got through' to practitioners. Social marketing techniques may help the promotion of evidence-based knowledge. This would entail systematically analysing and addressing barriers to clarity and acceptability of information, and offering a comprehensive and critical look at its validity, biases and relevance. However, paper does not fully describe or apply the key features of a social marketing approach.

Majdzadeh [32] 2008 Provide a conceptual framework to identify barriers and facilitators and design strategies to knowledge translation strategies to be used by organisations doing research	Five domains: Knowledge creation considers the characteristics of researchers and research Knowledge transfer considers resources and strategies Research utilization considers the characteristics of decision makers and context of decision making; Question transfer considers research priorities and funders Context of organization considers the leadership system, policies, values, and culture of the organisation doing research	**None stated** **Reference to other included frameworks** Jacobson Lavis	Practical Tehran University of Medical Sciences (TUMS) framework developed from review of literature Authors' suggest universities depend primarily on the passive dissemination of knowledge. They suggest the following strategies can make knowledge translation more effective in universities: defining and setting up of a system to assess the knowledge translation cycle; implementation and use of information technology; identification and encouragement of face-to-face interactions between researchers and decision makers; exchanging knowledgeable individuals among centres; creating mutual trust, a common language and culture for the creation of organizational knowledge; using important motivational tools in the university; using multidimensional methods for knowledge transfer

Friese [33] 2009 To identify what the cultural divides are between researchers and policymakers and how social scientists have bridged these differences by careful attention to several pragmatic practices for increasing research use in policymaking	Conceptualize policy work, not as disseminating information, but as developing relationships Take the initiative to contact policymakers or policy intermediaries Learn about the target policymaking audience Communicate research findings in ways that meet policymakers' information needs Use clear, careful language when dealing with myths about vulnerable populations Familiarize yourself with the policymaking process Provide a timely response to the questions driving the policy debate Learn how to approach policy work as an educator rather than an advocate Show respect for policymakers' knowledge and experience Be patient and self-rewarding in defining success.	**Two-communities theory** **Reference to other included frameworks** None	Based around notion that the underutilisation of research is down to a communication gap between researchers and policymakers, who have differing goals, information needs, values, and language that are best thought of as a cultural divide. Ten recommendations derived from qualitative interviews on the barriers and facilitators to research communication with social scientists working in family policy.

Yuan [34] 2010 Present a conceptual framework and propose a eight point strategy for improving the dissemination of best practices by national quality improvement campaigns	Provide simple, evidence- based recommendations Align messages with strategic goals of adopting organization Use a nodal organizational structure Engage a coalition of credible campaign sponsor Establish threshold of participating organizations Provide practical implementation tools Create networks to foster learning opportunities Monitor progress and evaluate impact	**Diffusion of innovations** Builds on Diffusion of Innovations but with a focus on active dissemination; planned efforts to persuade targeted groups to adopt an innovation **Reference to other included frameworks** Greenhalgh	Authors recognise that dissemination impact depends on contextual factors, including the nature of the innovation itself, external environmental incentives, and features of the adopting organizations. They argue that although important contextual considerations are outside the control of disseminators, greater use of their strategy is likely to promote more potent campaign efforts, more effective dissemination, and ultimately greater take-up of evidence-based practices.

### Theoretical underpinnings of dissemination frameworks

Thirteen of the twenty included dissemination frameworks were either explicitly or implicitly judged to be based on the Persuasive Communication Matrix [35,36]. Originally derived from a review of the literature of persuasion which sought to operationalise Lasswell's seminal description of persuasive communications as being about 'Who says what in which channel to whom with what effect' [37]. McGuire argued that there are five variables that influence the impact of persuasive communications. These are the source of communication, the message to be communicated, the channels of communication, the characteristics of the audience (receiver), and the setting (destination) in which the communication is received.

Included frameworks were judged to encompass either three [21,27,29], four [15,20,23,26,28,31,38], or all five [11,18,25] of McGuire's five input variables, namely, the source, channel, message, audience, and setting. The earliest conceptual model included in the review explicitly applied McGuire's five input variables to the dissemination of medical technology assessments [11]. Only one other framework (in its most recent version) explicitly acknowledges McGuire [17]; the original version acknowledged the influence of Winkler *et al*. on its approach to conceptualising systematic review dissemination [18]. The original version of the CRD approach [18,39] is itself referred to by two of the other eight frameworks [20,23]

Diffusion of Innovations theory [40,41] is explicitly cited by eight of the dissemination frameworks [11,17,19,22,24,28,29,34]. Diffusion of Innovations offers a theory of how, why, and at what rate practices or innovations spread through defined populations and social systems. The theory proposes that there are intrinsic characteristics of new ideas or innovations that determine their rate of adoption, and that actual uptake occurs over time via a five-phase innovation-decision process (knowledge, persuasion, decision, implementation, and confirmation). The included frameworks are focussed on the knowledge and persuasion stages of the innovation-decision process.

Two of the included dissemination frameworks make reference to Social Marketing [42]. One briefly discusses the potential application of social and commercial marketing and advertising principles and strategies in the promotion of non-commercial services, ideas, or research-based knowledge [22]. The other briefly argues that a social marketing approach could take into account a planning process involving 'consumer' oriented research, objective setting, identification of barriers, strategies, and new formats [30]. However, this framework itself does not represent a comprehensive application of social marketing theory and principles, and instead highlights five factors that are focussed around formatting evidence-based information so that it is clear and appealing by defined target audiences.

Three other distinct dissemination frameworks were included, two of which are based on literature reviews and researcher experience [14,32]. The first framework takes a novel question-based approach and aims to increase researchers' awareness of the type of context information that might prove useful when disseminating knowledge to target audiences [14]. The second framework presents a model that can be used to identify barriers and facilitators and to design interventions to aid the transfer and utilization of research knowledge [32]. The final framework is derived from Two Communities Theory [43] and proposes pragmatic strategies for communicating across conflicting cultures research and policy; it suggests a shift away from simple one-way communication of research to researchers developing collaborative relationships with policy makers [33].

### Characteristics of conceptual frameworks relating to knowledge translation that could be used by researchers to guide their dissemination activities

Table 2 summarises in chronological order the dissemination elements of 13 conceptual frameworks relating to knowledge translation that could be used by researchers to guide their dissemination activities [13,44-55].

**Table 2:** Conceptual frameworks relating to knowledge translation that could be used by researchers to guide their dissemination activities

Author, Year, Aims	Dissemination elements	Theoretical foundations	Description/Comment
Funk [44] 1989 To facilitate the use of research in clinical settings by providing findings that are relevant and ready to use, in a form that maintains the richness of full research reports yet is still understandable to the general reader.	Qualities of Research (described as topic selection based on literature reviews and surveys of clinicians with criteria focussed on relevance, applicability and the perceived gaps between evidence and practice) Characteristics of the communication (including use of non-technical language, emphasis on implications for practice and strategies for implementation). Facilitation of utilisation (provision of enquiry centre for implementation advice and to respond to requests for further information and feedback channel for researchers and practitioners)	**None stated** **Reference to other included frameworks** None	Describes an approach devised by the National Center for Nursing Research to make research results accessible to practising nurses via a topic focused conference and monograph series.

Lomas[12,45] 1993 Presents a coordinated implementation model that that seeks to shed light on dissemination processes and on best how to flow research findings into practice.	Dissemination elements within wider implementation model: The message Its source The communication channels The implementation setting	**Mixed** Full model derived from models of social influence, diffusion of innovations, adult learning theory and social marketing. **Persuasive communication** Four (source, setting, message, channel) of McGuire's five attributes of persuasive communication evident (explicitly derived from Winkler) **Reference to other included frameworks** Winkler	Argues that use of research in practice may depend more on a change in researchers behaviour than it does on practitioners-research findings most likely to find their way into practice when they are synthesised, contextualised, packaged to the needs of the end user. Wider model recognises the external influencing factors on the overall practice environment including, economic resources, legislation and regulation, education, personnel as well as public (media) and patient pressures.

Dobbins[13] 2002 To construct a comprehensive framework of research dissemination and utilisation.	Complex interrelationships that exist among five stages of innovation (knowledge, persuasion, decision, implementation and confirmation) and four types of characteristics (innovation, organization, environment and individual) as progression from research dissemination to research utilization occurs	**Diffusion of innovations** Explicit application of Rogers diffusion of innovations innovation-decision process **Reference to other included frameworks** None	Application of Rogers's innovation-decision process to health research dissemination and utilisation. Framework integrates concepts of research dissemination (knowledge, persuasion), evidence-based decision making (decision) and research utilisation (implementation) within the innovations decision process of diffusion of innovations theory. Argues that the extent to which an individual or organisation becomes knowledgeable about new ideas is somewhat dependent on the dissemination strategies employed by health researchers

Elliot [46] 2003 Present a conceptual and analytic frameworks that integrate several approaches to understanding and studying dissemination processes within public health systems focussed on cardiovascular health promotion	Four categories of factors shown to affect the success of dissemination efforts: Characteristics of the dissemination object Environmental factors, Factors associated with users Relationships between producers and users.	**Diffusion of innovations** Derived from Diffusion of Innovations-goes on to describe five approaches to dissemination (science push, problem solving, organisational, knowledge transfer and interaction) **Reference to other included frameworks** None	Authors state that dissemination and capacity exist within a broader social, political, economic context operating at micro, meso and macro levels The framework posits that contextual factors act as mediators shaping the behaviours and values of individuals and organizations, innovations, and influencing the process and outcome of capacity building and dissemination.

Greenhalgh [47,57] 2004 Review of the literature on the spread and sustainability of innovations in health service delivery and organisation Develop and apply (in four case studies) a unifying conceptual model based on the evidence.	Planned dissemination elements within wider model: Address needs and perspectives of potential adopters Tailor different strategies to different groups Use appropriate messages Use appropriate communication channels Undertake rigorous evaluation	**Diffusion of innovations** Application of Diffusion of Innovations in a health service delivery and organisation context **Persuasive communication** Not explicitly stated but four (message, setting, audience, channel) of McGuire's five attributes of persuasive communication **Reference to other included frameworks** None	Formal dissemination programs, defined as active and planned efforts to persuade target groups to adopt an innovation are more effective if the program's organizers (1) take full account of potential adopters' needs and perspectives, with particular attention to the balance of costs and benefits for them; (2) tailor different strategies to the different demographic, structural, and cultural features of different subgroups; (3) use a message with appropriate style, imagery, metaphors, and so on; (4) identify and use appropriate communication channels; and (5) incorporate rigorous evaluation and monitoring of defined goals and milestones

Green [48] 2006 Review tobacco control dissemination experience to draw guidance for physical activity promotion	Push: strengthening science push by proving, improving, and communicating effective interventions for wide population use; Pull: boosting demand, or market pull for interventions among consumers, and healthcare purchasers and policymakers Capacity: building the capacity of relevant systems and institutions to deliver them	**Diffusion of innovations** Diffusion of Innovations used to assess how tobacco control lessons diffuse and apply to the field of physical activity **Reference to other included frameworks** None	Author's state dissemination encompasses the planned facilitation and acceleration of diffusion of innovations, transfer and utilization of knowledge, and implementation of the resulting adaptations in local circumstances. Author suggest lessons from tobacco control include the need for a funded mandate; the mass media to frame the public policy debate and to help undermine negative behaviour; the comprehensiveness of interventions at national and local levels to mutually reinforce each other; the need for systematic evaluation; the need for policy and funding to support programs; the need for coordinated programs to support individuals.

Owen [49] 2006 Outline the main attributes of Diffusion of Innovations and key concepts to consider in the dissemination and diffusion of innovations to promote physical activity	Advocacy: identifying and engaging key stakeholders Increased funding to build the evidence base to supply diffusion and dissemination strategies and to allow investigators to gain experience with type of role Implement surveillance systems to track use of evidence-based interventions	**Diffusion of innovations** Application of Diffusion of Innovations in a public health context RE-AIM framework can be used to determine the success and impact of dissemination efforts **Reference to other included frameworks** None	Diffusion of innovations theory can be applied to accelerate the rate of diffusion specifically to promote physical activity interventions. Authors present two case studies and argue that their success illustrates the need for dedicated field staff, product production, marketing, and distribution.

Landry [50] 2007 To determine the extent of research transfer in natural sciences and engineering among Canadian university researchers; to examine any differences between various disciplines with regard to the extent of transfer; to examine the determinants of research transfer	Four categories of resources (along with the attributes of research knowledge) likely to enable researchers to transfer knowledge: Financial Organizational Relational Personal	**Resource-based view of the firm** Resource-based view of the firm-researchers have resources and capabilities which are deployed and mobilized in their knowledge transfer activities **Reference to other included frameworks** None	Based on a survey of 1,554 researchers, presents a model of how researchers in natural sciences and engineering transfer knowledge outside the academic community Two determinants found to be consistently influential: linkages between researchers and research users, and focus of the research projects on end user needs. Other determinants influencing knowledge transfer varied from one research field to another

Baumbusch [51] 2008 Describe a participatory approach to knowledge translation developed during a program of research concerning equitable care for diverse populations	Two dimensions process (translation) and content (knowledge): Process (translation involving: credible messengers, accountability, reciprocity, respect, and research champions) Content (ongoing cycle of data collection, analysis and synthesis of knowledge)	**None stated** **Reference to other included frameworks** Jacobson Lavis	A collaborative model of knowledge translation between researchers and practitioners in clinical settings-derived from a non systematic review of literature and from experiences drawn from a programme of research funded by the Canadian Institutes of Health Research. Authors state at the core of the approach is a collaborative relationship between researchers and practitioners, which underpins the knowledge translation cycle, and occurs simultaneously with data collection/analysis/synthesis

Feldstein [52] 2008 To provide a new tool for researchers and healthcare decision makers that integrates existing concepts relevant to translating research into practice.	Program or intervention (consideration of elements from the perspective of the organization and staff to be targeted) External environment (consideration of) Implementation and sustainability infrastructure necessary for success (consideration of) Recipients (Characteristics of both organisational and patient recipients of interventions need to be considered to maximize intervention effectiveness)	**Mixed** States that aspects of the model derived from diffusion of innovations, social ecology, the PRECEDE/PROCEED model, and the quality improvement/implementation literature. Impact measures derived from RE-AIM **Reference to other included frameworks** Jacobson Lavis	Practical, Robust Implementation and Sustainability Model (PRISM) considers how the program or intervention design, the external environment, the implementation and sustainability infrastructure, and the recipients influence program adoption, implementation, and maintenance. Designed to help researchers (and organisations) conceptualize, implement, and evaluate healthcare improvement programs.

Clinton [53] 2009 To present a knowledge transfer model and illustrate how its use can lead to competitive advantage	Comprehensive employee skills assessment Identify the type of knowledge to be transferred (tacit or explicit) Select appropriate media required for knowledge transfer Appropriate generation of corporate university (defined as a strategic commitment to organisational learning and development of intellectual capital)	**Knowledge-based view of the firm** **Reference to other included frameworks** None	The authors propose that the type of knowledge to be transferred and the appropriate media to transfer that knowledge, determine the education and training needs required to achieve competitive advantage

Mitchell [54] 2009 To identify dimensions that could be used to describe and differentiate models of partnerships, and illustrate how these dimensions could be applied using three recent case studies in Australia.	Decision maker involvement in research versus researcher involvement in decision making Investigator versus decision maker driven research Value of decision maker involvement at various stages of the research process. Discrete projects versus programs versus ongoing reciprocity Formal versus informal linkages Active versus passive involvement Concentrated and specific versus diffuse and heterogeneous linkages	**None stated** **Reference to other included frameworks** Greenhalgh Lavis	Dimensions derived from a brief narrative review of the partnership literature within health services research and on a selection of theoretical and conceptual references from other fields, particularly organization science. Authors argue building capacity for knowledge exchange demands an evidence-base of its own. They suggest their seven dimensions of partnerships provide a basis for research examining the usefulness of particular partnership models and their applicability and effectiveness in different contexts

Ward [55,56] 2009 Reviews knowledge transfer frameworks to gain a better understanding of the processes involved in knowledge transfer and presents a five domain model of the knowledge transfer processes to help researchers, practitioners and decision makers plan and evaluate initiatives for transferring knowledge into action	Problem: Identifying and communicating about the problem which the knowledge needs to address Context: Analysing the context which surrounds the producers and users of knowledge Knowledge: Developing and selecting the knowledge to be transferred Intervention: Selecting specific knowledge transfer activities or Interventions Use: Considering how the knowledge will be used in practice	**Mixed** Practical framework developed from on commonalities from 28 published models including the Diffusion of Innovations **Reference to other included frameworks** Dobbins Greenhalgh Jacobson Lavis	Authors emphasise that knowledge transfer is an interactive, multidirectional rather than linear process Report outlines a series of domain specific questions for research users and producers to use to think about and incorporate knowledge transfer processes in to their routine practice.

### Theoretical underpinnings of dissemination frameworks

Only two of the included knowledge translation frameworks were judged to encompass four of McGuire's five variables for persuasive communications [45,47]. One framework [45] explicitly attributes these variables as being derived from Winkler *et al *[11]. The other [47] refers to strong direct evidence but does not refer to McGuire or any of the other included frameworks.

Diffusion of Innovations theory [40,41] is explicitly cited in eight of the included knowledge translation frameworks [13,45-49,52,56]. Of these, two represent attempts to operationalise and apply the theory, one in the context of evidence-based decision making and practice [13], and the other to examine how innovations in organisation and delivery of health services spread and are sustained in health service organisations [47,57]. The other frameworks are exclusively based on the theory and are focussed instead on strategies to accelerate the uptake of evidence-based knowledge and or interventions

Two of the included knowledge translation frameworks [50,53] are explicitly based on resource or knowledge-based Theory of the Firm [58,59]. Both frameworks propose that successful knowledge transfer (or competitive advantage) is determined by the type of knowledge to be transferred as well as by the development and deployment of appropriate skills and infrastructure at an organisational level.

Two of the included knowledge translation frameworks purport to be based upon a range of theoretical perspectives. The Coordinated Implementation model is derived from a range of sources, including theories of social influence on attitude change, the Diffusion of Innovations, adult learning, and social marketing [45]. The Practical, Robust Implementation and Sustainability Model was developed using concepts from Diffusion of Innovations, social ecology, as well as the health promotion, quality improvement, and implementation literature [52].

Three other distinct knowledge translation frameworks were included, all of which are based on a combination of literature reviews and researcher experience [44,51,54].

### Conceptual frameworks provided by UK funders

Of the websites of the 10 UK funders of health services and public health research, only the ESRC made a dissemination framework available to grant applicants or holders (see Table 1) [26]. A summary version of another included framework is available via the publications section of the Joseph Rowntree Foundation [60]. However, no reference is made to it in the submission guidance they make available to research applicants.

All of the UK funding bodies made brief references to dissemination in their research grant application guides. These would simply ask applicants to briefly indicate how findings arising from the research will be disseminated (often stating that this should be other than via publication in peer-reviewed journals) so as to promote or facilitate take up by users in the health services.

## Discussion

This systematic scoping review presents to our knowledge the most comprehensive overview of conceptual/organising frameworks relating to research dissemination. Thirty-three frameworks met our inclusion criteria, 20 of which were designed to be used by researchers to guide their dissemination activities. Twenty-eight included frameworks that were underpinned at least in part by one or more of three different theoretical approaches, namely persuasive communication, diffusion of innovations theory, and social marketing.

Our search strategy was deliberately broad, and we searched a number of relevant databases and other sources with no language or publication status restrictions, reducing the chance that some relevant studies were excluded from the review and of publication or language bias. However, we restricted our searches to health and social science databases, and it is possible that searches targeting for example the management or marketing literature may have revealed additional frameworks. In addition, this review was undertaken as part of a project assessing UK research dissemination, so our search for frameworks provided by funding agencies was limited to the UK. It is possible that searches of funders operating in other geographical jurisdictions may have identified other studies. We are also aware that the way in which we have defined the process of dissemination and our judgements as to what constitutes sufficient detail may have resulted in some frameworks being excluded that others may have included or vice versa. Given this, and as an aid to transparency, we have included the list of excluded papers as Additional File 2, Appendix 2 so as to allow readers to assess our, and make their own, judgements on the literature identified.

Despite these potential limitations, in this review we have identified 33 frameworks that are available and could be used to help guide dissemination planning and activity. By way of contrast, a recent systematic review of the knowledge transfer and exchange literature (with broader aims and scope) [61] identified five organising frameworks developed to guide knowledge transfer and exchange initiatives (defined as involving more than one way communications and involving genuine interaction between researchers and target audiences) [13-15,62,63]. All were identified by our searches, but only three met our specific inclusion criteria of providing sufficient dissemination process detail [13-15]. One reviewed methods for assessment of research utilisation in policy making [62], whilst the other reviewed knowledge mapping as a tool for understanding the many knowledge creation and translation resources and processes in a health system [63].

There is a large amount of theoretical convergence among the identified frameworks. This all the more striking given the wide range of theoretical approaches that could be applied in the context of research dissemination [64], and the relative lack of cross-referencing between the included frameworks. Three distinct but interlinked theories appear to underpin (at least in part) 28 of the included frameworks. There has been some criticism of health communications that are overly reliant on linear messenger-receiver models and do not draw upon other aspects of communication theory [65]. Although researcher focused, the included frameworks appear more participatory than simple messenger-receiver models, and there is recognition of the importance of context and emphasis on the key to successful dissemination being dependent on the need for interaction with the end user.

As we highlight in the introduction, there is recognition among international funders both of the importance of and their role in the dissemination of research [9]. Given the current political emphasis on reducing deficiencies in the uptake of knowledge about the effects of interventions into routine practice, funders could be making and advocating more systematic use of conceptual frameworks in the planning of research dissemination.

Rather than asking applicants to briefly indicate how findings arising from their proposed research will be disseminated (as seems to be the case in the UK), funding agencies could consider encouraging grant applicants to adopt a theoretically-informed approach to their research dissemination. Such an approach could be made a conditional part of any grant application process; an organising framework such as those described in this review could be used to demonstrate the rationale and understanding underpinning their proposed plans for dissemination. More systematic use of conceptual frameworks would then provide opportunities to evaluate across a range of study designs whether utilising any of the identified frameworks to guide research dissemination does in fact enhance the uptake of research findings in policy and practice.

## Summary

There are currently a number of theoretically-informed frameworks available to researchers that could be used to help guide their dissemination planning and activity. Given the current emphasis on enhancing the uptake of knowledge about the effects of interventions into routine practice, funders could consider encouraging researchers to adopt a theoretically informed approach to their research dissemination.

## Competing interests

Paul Wilson is an Associate Editor of Implementation Science. All decisions on this manuscript were made by another senior editor. Paul Wilson works for, and has contributed to the development of the CRD framework which is included in this review. The author(s) declare that they have no other competing interests.

## Authors' contributions

All authors contributed to the conception, design, and analysis of the review. All authors were involved in the writing of the first and all subsequent versions of the paper. All authors read and approved the final manuscript. Paul Wilson is the guarantor.

## Supplementary Material

Additional file 1 **Appendix 1: Database search strategies**. This file includes details of the database specific search strategies used in the review. Click here for file

Additional file 2 **Appendix 2: Full-text papers assessed for eligibility but excluded from the review**. This file includes details of full-text papers assessed for eligibility but excluded from the review. Click here for file
