# Supplementary material for: Disseminating research findings: what should researchers do? A systematic scoping review of conceptual frameworks
Source: Implement Sci. 2010 Nov 22;5:91. doi: 10.1186/1748-5908-5-91 (PMC2994786; doi:10.1186/1748-5908-5-91)
Supplement: Additional file 1 — Appendix 1: Database search strategies. This file includes details of the database specific search strategies used in the review. [file 1748-5908-5-91-S1.DOC]

**Appendix 1: Database search strategies**

**MEDLINE and MEDLINE In-Process & Other Non-Indexed Citations (OvidSP)**

**1950-2010/June week 1**

1. *"Diffusion of Innovation"/ or *Information Dissemination/ or *Information Management/

2. exp *Research Design/ or exp *Health Services Research/ or *Evidence-Based Medicine/

3. 1 and 2

4. (knowledge adj1 (transfer or translation or generation or uptake or exchange or broker$ or mobili?ation or utili?ation or implementation or adopt$)).ti,ab.

5. (evidence adj1 (transfer or translation or generation or uptake or exchange or broker$ or mobili?ation or utili?ation or implementation or adopt$)).ti,ab.

6. (diffus$ adj2 (innovation or information)).ti,ab.

7. (disseminat$ adj3 (research or framework or evidence)).ti,ab.

8. research into practice.ti,ab.

9. research findings into practice.ti,ab.

10. evidence into practice.ti,ab.

11. or/3-10

12. exp animals/ not (exp animals/ and humans/)

13. 11 not 12

**EMBASE (OvidSP) 1980-2010/week 22**

1. (knowledge adj1 (transfer or translation or generation or uptake or exchange or broker$ or mobili?ation or utili?ation or implementation or adopt$)).ti,ab.

2. (evidence adj1 (transfer or translation or generation or uptake or exchange or broker$ or mobili?ation or utili?ation or implementation or adopt$)).ti,ab.

3. (diffus$ adj2 (innovation or information)).ti,ab.

4. (disseminat$ adj3 (research or framework or evidence)).ti,ab.

5. research into practice.ti,ab.

6. research findings into practice.ti,ab.

7. evidence into practice.ti,ab.

8. or/1-7

9. Animal/ or Animal Experiment/ or Nonhuman/

10. (rat or rats or mouse or mice or murine or rodent or rodents or hamster or hamsters or pig or pigs or porcine or rabbit or rabbits or animal or animals or dogs or dog or cats or cow or bovine or sheep or ovine or monkey or monkeys).ti,ab,sh.

11. 9 or 10

12. exp Human/ or Human Experiment/

13. 11 not (11 and 12)

14. 8 not 13

**CINAHL (EBSCO) 1981-2010/June week 1**

S1 (MH "Study Design") OR (MH "Health Services Research")

S2 (MH "diffusion of innovation")

S3 S1 and S2

S4 TI (knowledge N1 transfer or knowledge N1 translation or knowledge N1 generation or knowledge N1 uptake or knowledge N1 exchange or knowledge N1 broker* or knowledge N1 mobilization or knowledge N1 mobilisation or knowledge N1 utilization or knowledge N1 utilization or knowledge N1 implementation or knowledge N1 adopt*) OR AB (knowledge N1 transfer or knowledge N1 translation or knowledge N1 generation or knowledge N1 uptake or knowledge N1 exchange or knowledge N1 broker* or knowledge N1 N1 mobilisation or knowledge N1 utilization or knowledge N1 utilization or knowledge N1 implementation or knowledge N1 adopt*)

S5 TI (evidence N1 transfer or evidence N1 translation or evidence N1 generation or evidence N1 uptake or evidence N1 exchange or evidence N1 broker* or evidence N1 mobilization or evidence N1 mobilisation or evidence N1 utilization or evidence N1 utilization or evidence N1 implementation or evidence N1 adopt*) OR AB (evidence N1 transfer or evidence N1 translation or evidence N1 generation or evidence N1 uptake or evidence N1 exchange or evidence N1 broker* or evidence N1 mobilization or evidence N1 utilization or evidence N1 utilization or evidence N1 implementation or evidence N1 adopt*)

S6 TI (diffus* N2 innovation or diffus* N2 information) OR AB (diffus* N2 innovation or diffus* N2 information)

S7 TI (disseminat* N3 research or disseminat* N3 framework or disseminat* N3 evidence) OR AB (disseminat* N3 research or disseminat* N3 framework or disseminat* N3 evidence)

S8 S3 or S4 or S5 or S6 or S7

**PsycINFO (OivdSP) 1806-2010/June week 1**

1. Knowledge Transfer/

2. Information Dissemination/

3. (knowledge adj1 (transfer or translation or generation or uptake or exchange or broker$ or mobili?ation or utili?ation or implementation or adopt$)).ti,ab.

4. (evidence adj1 (transfer or translation or generation or uptake or exchange or broker$ or mobili?ation or utili?ation or implementation or adopt$)).ti,ab.

5. (diffus$ adj2 (innovation or information)).ti,ab.

6. (disseminat$ adj3 (research or framework or evidence)).ti,ab.

7. research into practice.ti,ab.

8. research findings into practice.ti,ab.

9. evidence into practice.ti,ab.

10. or/1-9

**EconLit (OivdSP) 1969-2010/June**

1. (knowledge adj1 (transfer or translation or generation or uptake or exchange or broker$ or mobili?ation or utili?ation or implementation or adopt$)).ti,ab.

2. (evidence adj1 (transfer or translation or generation or uptake or exchange or broker$ or mobili?ation or utili?ation or implementation or adopt$)).ti,ab.

3. (diffus$ adj2 (innovation or information)).ti,ab.

4. (disseminat$ adj3 (research or framework or evidence)).ti,ab.

5. research into practice.ti,ab.

6. research findings into practice.ti,ab.

7. evidence into practice.ti,ab.

8. or/1-7

**Social Services Abstracts (CSA Illumina) 1979-2010/June**

(TI=((knowledge) within 1 (transfer or translation or generation or uptake or exchange or broker* or mobilization or mobilization or utilization or utilization or implementation or adopt*)) or AB=((knowledge) within 1 (transfer or translation or generation or uptake or exchange or broker* or mobilization or mobilization or utilization or utilization or implementation or adopt*))) or(TI=((evidence) within 1 (transfer or translation or generation or uptake or exchange or broker* or mobilization or mobilization or utilization or utilization or implementation or adopt*)) or AB=((evidence) within 1 (transfer or translation or generation or uptake or exchange or broker* or mobilization or mobilization or utilization or utilization or implementation or adopt*))) or(TI=((diffus*) within 2 (innovation or information)) or AB=((diffus*) within 2 (innovation or information))) or(TI=("research into practice") or AB=("research into practice")) or(TI=("research findings into practice") or AB=("research findings into practice")) or(TI=("evidence into practice") or AB=("evidence into practice"))

**Social Policy & Practice (OvidSP) 1890-2010/June**

1. (knowledge adj1 (transfer or translation or generation or uptake or exchange or broker$ or mobili?ation or utili?ation or implementation or adopt$)).ti,ab.

2. (evidence adj1 (transfer or translation or generation or uptake or exchange or broker$ or mobili?ation or utili?ation or implementation or adopt$)).ti,ab.

3. (diffus$ adj2 (innovation or information)).ti,ab.

4. (disseminat$ adj3 (research or framework or evidence)).ti,ab.

5. or/1-4

**CDSR, CENTRAL, CMR, DARE, HTA and NHS EED (Cochrane Library)**

**2010: Issue 1**

#1 MeSH descriptor Diffusion of Innovation explode all trees

#2 MeSH descriptor Information Dissemination explode all trees

#3 MeSH descriptor Information Management explode all trees

#4 (#1 OR #2 OR #3)

#5 MeSH descriptor Research Design explode all trees

#6 MeSH descriptor Health Services Research explode all trees

#7 MeSH descriptor Evidence-Based Medicine explode all trees

#8 (#5 OR #6 OR #7)

#9 (#4 AND #8)

#10 (knowledge NEAR/1 (transfer or translation or generation or uptake or exchange or broker* or mobili?ation or utili?ation or implementation or adopt*)):ti,ab

#11 (evidence NEAR/1 (transfer or translation or generation or uptake or exchange or broker* or mobili?ation or utili?ation or implementation or adopt*)):ti,ab

#12 (diffus* NEAR/2 (innovation or information)):ti,ab

#13 (disseminat* NEAR/3 (research or framework or evidence)):ti,ab

#14 "research into practice":ti,ab

#15 "research findings into practice":ti,ab

#16 "evidence into practice":ti,ab

#17 (#9 OR #10 OR #11 OR #12 OR #13 OR #14 OR #15 OR #16)
