# Supplementary material for: Disseminating research findings: what should researchers do? A systematic scoping review of conceptual frameworks
Source: Implement Sci. 2010 Nov 22;5:91. doi: 10.1186/1748-5908-5-91 (PMC2994786; doi:10.1186/1748-5908-5-91)
Supplement: Additional file 2 — Appendix 2: Full-text papers assessed for eligibility but excluded from the review. This file includes details of full-text papers assessed for eligibility but excluded from the review. [file 1748-5908-5-91-S2.DOC]

**Appendix 2: Full-text papers assessed for eligibility but excluded from the review.**

Addis ME. Methods for disseminating research products and increasing evidence-based practice: Promises, obstacles, and future directions. *Clin Psychol Sci Pract* 2002; 9:367-378.

Aita M, Richer M-C, Heon M. Illuminating the processes of knowledge transfer in nursing. *Worldviews Evid Based Nurs* 2007; 4:146-55.

Ament LA. Strategies for dissemination of policy research. *J Nurse Midwifery* 1994; 39:329-31.

Armstrong R, Waters E, Roberts H, Oliver S, Popay J. The role and theoretical evolution of knowledge translation and exchange in public health. *J Public Health* 2006; 28:384-9.

Arai L, Roen K, Roberts H, Popay J. It might work in Oklahoma but will it work in Oakhampton? Context and implementation in the effectiveness literature on domestic smoke detectors. *Inj Prev* 2005; 11:148–51.

Backer TE, Liberman RP, Kuehnel TG. Dissemination and adoption of innovative psychosocial interventions. *J Consult Clin Psychol* 1986; 54:111-118.

Baker EA, Brennan Ramirez LK, Claus JM, Land G. Translating and disseminating research- and practice-based criteria to support evidence-based intervention planning. *J Public Health Manage Pract* 2008; 14:124-30.

Bannigan K. Disseminating research results. *Ment Health Occup Ther* 2005; 10:88-9.

Barg FK, McCorkle R, Robinson K, Yasko JM, Jepson C, McKeehan KM. Gaps and contract: evaluating the diffusion of new information. Part I. A description of the strategy. *Cancer Nurs* 1992; 15:401-5.

Battista RN. Innovation and diffusion of health-related technologies. A conceptual framework. *Int J Technol Assess in Health Care* 1989; 5:227-48.

Berwick DM. Disseminating innovations in health care. *JAMA* 2003; 289:1969-75.

Best A, Moor G, Holmes B, Clark PI, Bruce T, Leischow S, Buchholz K, Krajnak J. Health promotion dissemination and systems thinking: towards an integrative model. *Am J Health Behav* 2003; 27 (Suppl 3):S206-16.

Bou-Llusar JC, Segarra-Cipres M. Strategic knowledge transfer and its implications for competitive advantage: An integrative conceptual framework. *Journal of Knowledge Management* 2006; 10:100-112.

Bowen S, Zwi AB. Pathways to "evidence-informed" policy and practice: A framework for action. *Plos Med* 2005; 2:600-605.

Bowen DJ, Sorensen G, Weiner BJ, Campbell M, Emmons K, Melvin C. Dissemination research in cancer control: where are we and where should we go? *Cancer Causes Control* 2009; 20:473-85.

Brachos D, Kostopoulos K, Soderquist KE, Prastacos G. Knowledge effectiveness, social context and innovation. *Journal of Knowledge Management* 2007; 11:31-44.

Bradley EH, Webster TR, Baker D, Schlesinger M, Inouye SK, Barth MC, Lapane KL, Lipson D, Stone R, Koren MJ. Translating research into practice: speeding the adoption of innovative health care programs. *Issue Brief (Commonw Fund)*. 2004; 724:1-12.

Brown GT, Rodger S. Research utilization models: frameworks for implementing evidence-based occupational therapy practice. *Occup Ther Int* 1999; 6:1-23.

Bucknall T. Knowledge transfer and utilization: implications for home healthcare pain management. *J Healthc Qual* 2006; 28:12-9.

Chunharas S. An interactive integrative approach to translating knowledge and building a "learning organization" in health services management. *Bull World Health Organ* 2006; 84:652-7.

Conklin J, Stolee P. A model for evaluating knowledge exchange in a network context. *Can J Nurs Res* 2008;40:116-24.

Cronenwett LR. Effective methods for disseminating research findings to nurses in practice. *Nurs Clin North Am* 1995;30:429-38.

Crosswaite C, Curtice L. Disseminating research results - The challenge of bridging the gap between health research and health action. *Health Promot Int* 1994; 9:289-296.

Davis SM, Peterson JC, Helfrich CD, Cunningham-Sabo L. Introduction and conceptual model for utilization of prevention research. *Am J Prev Med* 2007; 33(1 Suppl):S1-S5.

Dearing JW. Evolution of diffusion and dissemination theory. *J Public Health Manage Pract* 2008; 14:99-108.

Dearing JW, Maibach EW, Buller DB. A convergent diffusion and social marketing approach for disseminating proven approaches to physical activity promotion. *Am J Prev Med* 2006; 31(4 Suppl):S11-23.

Dobbins M, DeCorby K, Twiddy T. A knowledge transfer strategy for public health decision makers. *Worldviews Evid Based Nurs* 2004; 1:120-8.

Ebener S, Khan A, Shademani R, Compernolle L, Beltran M, Lansang M, Lippman M. Knowledge mapping as a technique to support knowledge translation. *Bull World Health Organ* 2006; 84:636-42.

Eccles M, Grimshaw J, Walker A, Johnston M, Pitts N. Changing the behavior of healthcare professionals: the use of theory in promoting the uptake of research findings. *J Clin Epidemiol* 2005; 58:107-12.

Elliott SJ, O'Loughlin J, Robinson K, Eyles J, Cameron R, Harvey D, Raine K, Gelskey D; Canadian Heart Health Dissemination Project Strategic and Research Advisory Groups. Conceptualizing dissemination research and activity: the case of the Canadian Heart Health Initiative. *Health Educ Behav* 2003; 30:267-86.

Elwyn G, Taubert M, Kowalczuk J. Sticky knowledge: A possible model for investigating implementation in healthcare contexts. *Implement Sci* 2007;2:44.

Emshoff JG. Researchers, practitioners, and funders: using the framework to get us on the same page. *Am J Community Psychol* 2008; 41:393-403.

Estabrooks CA, Thompson DS, Lovely JJ, Hofmeyer A. A guide to knowledge translation theory. *J Contin Educ Health Prof* 2006; 26:25-36.

Ginsburg LR, Lewis S, Zackheim L, Casebeer A. Revisiting interaction in knowledge translation. *Implement Science* 2007;2:34.

Goering P, Butterill D, Jacobson N, Sturtevant D. Linkage and exchange at the organizational level: a model of collaboration between research and policy. *J Health Serv Res Policy* 2003; 8(Suppl 2):14-9.

Graham ID, Tetroe J, KT Theories Research Group. Some theoretical

underpinnings of knowledge translation. *Acad Emerg Med* 2007; 14:936-41.

Haines A, Kuruvilla S, Borchert M. Bridging the implementation gap between knowledge and action for health. *Bull World Health Organ* 2004; 82:724-31.

Hanney S, Gonzalez-Block M, Buxton M, Kogan M. The utilisation of health research in policy-making: concepts, examples and methods of assessment. *Health Res Policy Syst* 2003; 1:2.

Ho K, Bloch R, Gondocz T, Laprise R, Perrier L, Ryan D, Thivierge R, Wenghofer E. Technology-enabled knowledge translation: frameworks to promote research and practice. *J Contin Educ Health Prof* 2004; 24:90-9.

Institute of Health Economics. Effective dissemination of findings from research: a compilation of essays. Edmonton, AB: *Institute of Health Economics;* 2008.

Jacobson N, Butterill D, Goering P. Organizational factors that influence university-based researchers' engagement in knowledge transfer activities. *Sci Commun* 2004; 25:246-259.

Jacobson N, Butterill D, Goering P. Consulting as a strategy for knowledge transfer. *Milbank Q* 2005; 83:299-321.

Jacobson N, Goering P. Credibility and credibility work in knowledge transfer. *Evidence and Policy* 2006; 2:151-165.

Jacobson N, Ochocka J, Wise J, Janzen R, Taking Culture Seriously Partners. Inspiring knowledge mobilization through a communications policy: the case of a community university research alliance. *Prog Community Health Partnersh* 2007; 1:99-104.

Jensen RJ, Szulanski G. Template use and the effectiveness of knowledge transfer. *Manage Sci* 2007; 53:1716-30.

Jones R, Spencer J. Conference report: teaching old docs new tricks: research dissemination and professional behavioural change. *Fam Pract* 1993; 10:229-30.

Jones RJE, Santaguida P. Evidence-based practice and health policy development: the link between knowledge and action. *Physiotherapy* 2005; 91:14-21.

Kanouse DE, Kallich JD, Kahan JP. Dissemination of effectiveness and outcomes research. *Health Policy* 1995; 34:167-192.

Kitson A, Ahmed LB, Harvey G, Seers K, Thompson DR. From research to practice: one organizational model for promoting research-based practice. *J Adv Nurs* 1996; 23:430-40.

Kouri D. Knowledge exchange strategies for interventions and policy in public health. *Evidence and Policy* 2009; 5:71-83.

Kreuter MW, Bernhardt JM. Reframing the dissemination challenge: a marketing and distribution perspective. *Am J Public Health* 2009; 99:2123-7.

Landry R, Amara N, Pablos-Mendes A, Shademani R, Gold I. The knowledge-value chain: A conceptual framework for knowledge translation in health. *Bull World Health Organ* 2006; 84:597-602.

Lankshear S, Brierley JD, Imrie K, Yurcan M. Changing physician practice: an evaluation of knowledge transfer strategies to enhance physician documentation of cancer stage. *Healthc Q* 2010; 13:84-92.

Lawrence R. Research dissemination: actively bringing the research and policy worlds together. *Evidence and Policy* 2006; 2:373-384

Lomas J. Finding audiences, changing beliefs: the structure of research use in Canadian health policy. *J Health Polit Policy Law* 1990; 15:525-42.

Lomas J. Words without Action - the Production, Dissemination, and Impact of Consensus Recommendations. *Annual Review of Public Health* 1991;12:41-65.

Mendel P, Meredith LS, Schoenbaum M, Sherbourne CD, Wells KB. Interventions in organizational and community context: a framework for building evidence on dissemination and implementation in health services research. *Adm Policy Ment Health* 2008; 35:21-37.

Mitton C, Adair CE, McKenzie E, Patten SB, Waye Perry B. Knowledge transfer and exchange: review and synthesis of the literature. *Milbank Q* 2007; 85:729-68.

Moulding NT, Silagy CA, Weller DP. A framework for effective management of change in clinical practice: dissemination and implementation of clinical practice guidelines. *Qual Health Care* 1999; 8:177-83.

Nutbeam D, Boxall AM. What influences the transfer of research into health policy and practice? Observations from England and Australia. *Public Health* 2008; 122:747-53.

Nutley S, Davies HTO. Making a reality of evidence-based practice: some lessons from the diffusion of innovations. *Public Money Manage* 2000; 20:35-42.

Nutley S, Walter I, Davies HT. From Knowing to Doing: A Framework for Understanding the Evidence-Into-Practice Agenda. *Evaluation: The International Journal of Theory, Research and Practice* 2003; 9:125-148.

Osterling KL, Austin MJ. The dissemination and utilization of research for promoting evidence-based practice. *J Evid Based Soc Work* 2008; 5:295-319.

Ottoson JM. Knowledge-for-Action Theories in Evaluation: Knowledge Utilization, Diffusion, Implementation, Transfer, and Translation. *New Directions for Evaluation* 2009; 124:7-20.

Pang T, Sadana R, Hanney S, Bhutta ZA, Hyder AA, Simon J. Knowledge for better health: a conceptual framework and foundation for health research systems. *Bull World Health Organ* 2003; 81:815-20.

Pronovost PJ, Berenholtz SM, Needham DM. Translating evidence into practice: a model for large scale knowledge translation. *BMJ* 2008; 337:a1714.

Rabin BA, Brownson RC, Haire-Joshu D, Kreuter MW, Weaver NL. A glossary for dissemination and implementation research in health. *J Public Health Manage Pract* 2008;14:117-23.

Richardson A, Jackson C, Sykes W. *Taking research seriously: means of improving and assessing the use and dissemination of research*. London: HMSO; 1990.

Schwamm L, Fayad P, Acker JE 3rd, Duncan P, Fonarow GC, Girgus M, Goldstein LB, Gregory T, Kelly-Hayes M, Sacco RL, Saver JL, Segrest W, Solis P, Yancy CW. Translating evidence into practice: a decade of efforts by the American Heart Association/American Stroke Association to reduce death and disability due to stroke: a presidential advisory from the American Heart Association/American Stroke Association. *Stroke* 2010; 41:1051-65

Shinn E, Basen-Engquist K, Crain B, Follen M. A theory-aided dissemination strategy for emerging technologies in cervical cancer screening. *Gynecol Oncol* 2007; 107(1 Suppl):S35-S39.

Stirman SW, Crits-Christoph P, DeRubeis RJ. Achieving successful dissemination of empirically supported psychotherapies: A synthesis of dissemination theory. Clin Psychol Sci Pract 2004; 11:343-59.

Swinburn B, Gill T, Kumanyika S. Obesity prevention: a proposed framework for translating evidence into action. *Obes Rev* 2005; 6:23–33.

Tenove SC. Dissemination: current conversations and practices. *Can J Nurs Res* 1999; 31:95-9.

Tetroe JM, Graham ID, Foy R, Robinson N, Eccles MP, Wensing M, Durieux P, Légaré F, Nielson CP, Adily A, Ward JE, Porter C, Shea B, Grimshaw JM. Health research funding agencies' support and promotion of knowledge translation: an international study. *Milbank Q* 2008; 86:125-55.

Thompson GN, Estabrooks CA, Degner LF. Clarifying the concepts in knowledge transfer: a literature review. *J Adv Nurs* 2006; 53:691-701.

Tugwell P, Robinson V, Grimshaw J, Santesso N. Systematic reviews and knowledge translation. *Bull World Health Organ* 2006; 84:643-51.

Wandersman A, Duffy J, Flaspohler P, Noonan R, Lubell K, Stillman L, Blachman M, Dunville R, Saul J. Bridging the gap between prevention research and practice: the interactive systems framework for dissemination and implementation. *Am J Community Psychol* 2008; 41:171-81.

Wang S, Moss JR, Hiller JE. Applicability and transferability of interventions in evidence-based public health. *Health Promot Int* 2006; 21:76–83.
